# Etiology and Clinical Characteristics of Influenza-Like Illness (ILI) in Outpatients in Beijing, June 2010 to May 2011

**DOI:** 10.1371/journal.pone.0028786

**Published:** 2012-01-04

**Authors:** XiaoHua Yang, Yao Yao, MeiFang Chen, Xia Yang, YanDi Xie, YaFen Liu, XiuYing Zhao, Yan Gao, Lai Wei

**Affiliations:** 1 Department of Infectious Disease, Peking University Hepatology Institute, Peking University People's Hospital, Beijing, China; 2 Center of Clinical Lab, Beijing YouAn Hospital, Capital Medical University, Beijing, China; University of Hong Kong, Hong Kong

## Abstract

**Background:**

Since May 2009, exposure of the population of Beijing, China to pH1N1 has resulted in an increase in respiratory illnesses. Limited information is available on the etiology and clinical characteristics of the influenza-like illness (ILI) that ensued in adults following the pH1N1 pandemic.

**Methods:**

Clinical and epidemiological data of ILI in adults was collected. A total of 279 throat swabs were tested for twelve respiratory viruses using multiplex RT-PCR. Clinical characteristics of influenza A in outpatients versus test-negative patients were compared using Pearson's χ2 and the Mann-Whitney U test. 190 swabs were tested for pH1N1 by virus isolation. Consultation rates for ILI were compared between 2009 and 2010.

**Results:**

One or two virus were detected in 29% of the samples. Influenza A virus (FLU-A) accounted for 22.9% (64/279). Other viruses were present at a frequency less than 3.0%. Cough was significantly associated with Influenza A virus infection (χ2, p<0.001). The positive rate of FLU-A was consistent with changes in the ILI rate during the same period and there was a significant reduction in the incidence of ILI in 2010 when compared to 2009. During the 2010–2011 influenza season, the incidence peaked in January 2011 in Beijing and north China.

**Conclusions:**

Exposure to pH1N1 had no impact on typical influenza seasonal peaks, although FLU-A remained the predominant virus for 2010 in Beijing. Symptomatically, cough was associated with FLU-A infection. The positive rate of influenza virus was consistent with changes in the ILI rate during the same period and there was a significant reduction in the incidence of ILI in 2010 when compared to that of 2009.

## Introduction

Influenza virus is a seasonal acute respiratory infection and is associated with significant morbidity and mortality in adults every winter [1]. Similar to most other countries, the peak influenza season in China is associated with higher health care utilization [2]. In China, weekly cases of influenza-like illness (ILI) in acute-care settings throughout the country are reported to the Chinese National Influenza Center(CNIC) and are mainly attributed to influenza virus, predominantly influenza A virus (FLU-A) and influenza B virus (FLU-B) [3], [4]. In addition to the influenza viruses, other respiratory viruses can also cause ILI symptoms in adults, such as human respiratory syncytial virus (HRSV), human parainfluenza viruses (HPIV), including parainfluenza virus type 4 [5], rhinovirus, adenovirus, human coronaviruses and human metapneumovirus [6]. Thus, multiple respiratory viruses may circulate among adults and cause influenza symptoms [5]. There is inconsistency about whether certain symptoms can be used to distinguish specific infections, and it is generally accepted that there are no symptoms specific to any viral infection. Some studies have attempted to identify signs or symptoms specifically associated with influenza virus or other viruses [7], [8], but no definitive conclusions have been drawn.

ILI data such as ILI rate and ILI count can to some extent reflect influenza activity, and ILI attack rates may be higher among adults during pandemics [9]. In China, ILI data from larger hospitals can deliver valuable information that could be used for monitoring the onset of an epidemic [3]. Peking University People's Hospital (PKUPH), an affiliated and teaching hospital of Peking University, is a nonprofit health care institution that provides an exceptionally caring environment for patients and families, and extends care to patients of any nationality. PKUPH receives approximately 2,000,000 outpatient cases annually and patients from areas beyond Beijing (mainly northern China) account for forty percent of this figure. The Infectious Diseases Department of PKUPH is a national influenza surveillance sentinel unit.

Some studies have focused on changes in ILI when pH1N1 is present. However, few studies have reported on whether there have been changes in viral etiology and distribution, as well as clinical and epidemiological characteristics of ILI, following a pH1N1 pandemic. In this study, we focused on the etiology and epidemiological characteristics of adults with ILI symptoms after the 2009 influenza (H1N1) pandemic in Beijing.

## Materials and Methods

### Ethics statement

This research study did not involve any health-related patient interventions. The study was conducted in accordance with the principles and guidelines expressed in the Declaration of Helsinki and was approved as less than minimal risk research by the Research Ethics Committee at PKUPH. Written consent forms were not required and approved information sheets were used instead of consent forms. Detailed information was given to all patients, and once informed consent had been received, swab samples were collected and the data analyzed anonymously.

### Case definition and study population

A human influenza-like illness (ILI) case was clinically defined as a sudden onset of fever (≥38°C), cough and/or sore throat of less than three days duration, and no laboratory-confirmed evidence of another etiology [3], [10]. ILI cases were defined as FLU-A-positive ILI-cases when the RT-PCR results from throat swab samples were positive for influenza A virus. A co-infection was diagnosed when more than one virus tested positive on RT-PCR. The study population included adults (≥16 years) who sought medical attention in the fever outpatient service of PKUPH, a national influenza surveillance sentinel unit, between June 2010 and May 2011.

### Clinical specimens and data collection

Data was collected at the time of medical consultation using a case report form for each adult patient who met the ILI-case definition criteria. Data collected included demography, clinical observations (such as symptoms, chronic medical conditions, treatment, temperature) and laboratory test results. For RT-PCR-positive patients, we additionally used telephone interviews to determine treatment, co-morbidity, vaccination status and duration of fever. All variables and analyzed data are shown in **Table 1**. A single throat swab, using Sterile Rayon Swabs( 167KS01), was collected from each ILI-patient who had not received antiviral drugs. The swab was immediately placed in virus transport media tubes. Each sample was frozen within 24 hours at −70°C until analyzed. In total, 279 clinical specimens were analyzed.

**Table 1:** Clinical characteristics of ILI-patients in PKU People' Hospital between June 2010 and May 2011.

Characteristics		FLU-A-positive ILI-case	Other virus-positive ILI-case*	Virus-negative ILI-case	Total##
		(n = 69)	(n = 13)	(n = 197)	(n = 279)
Demographics					
	Age(years)	32(27–48)	28(22–40)	30(26–43)(n = 197)	31(26–44)
	Male	30(43.5%)	8(61.5%)	105(53.3%)	143(51.3%)
Clinical presentation					
	Temperature(°C)	38.6[38.4,38.7](n = 64)	38.8[38.3,39.0](n = 10)	38.6[38.5,38.7](n = 158)	38.6[38.5,38.6](n = 232)
	Temperature(°C)>39°C	17(24.6% of 69)	4(30.8% of 13)	63(32.1% of 196)	84(30.2% of 279)
	Cough	48(78.7% of 61)	10(76.9%)	75(50.3% of 149)	133(59.6% of 223)
	Sore throat	41(67.2% of 61)	11(84.6%)	88(59.1% of 149)	140(62.8% of 223)
	Headache	41(67.2% of 61)	7(53.8%)	92(61.7% of 149)	140(62.8% of 223)
	Myalgia	38(62.3% of 61)	10(76.9%)	98(65.8% of 149)	146(65.5% of 223)
	Diarrhea	1(1.6% of 61)	0(0%)	5(3.4% of 149)	6(2.7% of 223)
Symptoms onset (days)		1(1–2)(n = 65)	1.5(1–2)	1(1–2)(n = 190)	1(1–2)(n = 268)
routine examination					
	White cell count(×10^9^/l)	7.9(6.2–9.7)(n = 64)	9.6(7.9–13.5)(n = 10)	9.0(6.0–11.5)(n = 158)	8.3(6.2–11.2)(n = 232)
	Percentage of neutrophils(%)	77.6[75.4,79.7](n = 64)	76.1[68.6,83.7](n = 10)	77.8[76.6,79.5](n = 158)	77.6[76.3,79.0](n = 232)
	N%<70%	9(14.1% of 64)	3(30% of 10)	29(18.4% of 158)	41(17.7% of 232)
Other**					
	Vaccination history	8(18.6% of 43)	3(33.3% of 9)	0(0% of 42)	11(11.7% of 94)
	Comorbidity	7(11.7% of 60)	1(9.1% of 11)	11(12.8% of 86)	19(12.1% of 157)
	Antivirus treatments	4(19.0% of 21)	0(0% of 4)		4(14.8% of 27)
	Duration of fever(days)	4(3–5)(n = 33)	4(3–5)(n = 9)		4(3–5)(n = 44)
	Systemic symptom disappeared(days)	4(3–7)(n = 32)	3(2–6)(n = 9)		4(3–7)(n = 43)

Values are median (IQR) or n (%) of patients unless otherwise stated. Normally distributed data are reported as means with 95% CI and non-normally distributed data as medians with interquartile range.

The characteristics contain demography characteristics, medical history, presenting symptoms, and clinical findings.

*Positive cases for at least one respiratory virus except Influenza A viruses by RT-PCT.

**Telephone follow-up data mainly for RT-PCR-positive patients.

## Includes all ILI cases regardless of virology RT-PCR test results.

### Laboratory investigations

#### Viral RNA extraction and PCR amplification

Viral RNA was extracted from 140 µl of viral transport medium containing the swabs, using the QIAamp Viral RNA Mini Kit (Qiagen Viral RNA Mini Extraction Kit, Germany), following the manufacturer's instructions. Extracted RNA was then used as the template to perform the RT-PCR reaction, using a reverse-transcriptase polymerase chain reaction (RT-PCR) kit (Fermentas, Shenzhen). cDNA was compounded under the action of reverse transcriptase and random primers from RNA. The RT-PCR amplification system was as follows: total RNA 8 µl, random primers 1 µl, 5×RT buffer 4 µl, 10 mM dNTP 2 µl, RNA inhibitor 1 µl, and 1 µl reverse transcriptase added to DEPC treated water to 20 µl. Conditions used for the two-step RT-PCR were as follows: the primary RT-PCR was incubated at 80°C for 3 minutes, cooled down on ice for 2 minutes; followed by 37°C for 90 minutes and 94°C for 2 minutes, then cooled down on ice for a further 2 minutes, and stored at −20°C until collected.

#### Testing the virus using multiplex PCR

RT-PCR products were tested as per manufacturer's instructions for the following viruses: influenza A and B virus; respiratory syncytial virus (RSV); human coronaviruses 229E, OC43, NL63, HKU1; parainfluenza virus; enterovirus or rhinoviruses; adenoviruses; and finally metapneumovirus, using the 12 league respiratory virus multiple PCR detection kit (Neuro-Hemin Hangzhou, RV1211). The PCR amplification system was as follows: cDNA templates 3 µl, 5×RV Primer 4 µl, 8-Mop Solution 3 µl, and 2×Multiplex Master Mix 10 µl. PCR was carried out in a PCR Amplifier and the conditions were as follows: 94°C for 15 minutes, followed by 40 cycles of 94°C for 0.5 minutes, 60°C for 1.5 minutes and 72°C for 1.5 minutes, with extension at 72°C for 10 minutes, followed by storage at 4°C until collected. The samples were divided into two groups for testing, Group A samples were tested for the presence of adenovirus (Adv), metapneumovirus (MPV), human coronavirus 229E/NL63 and parainfluenza virus (PIV) types 1, 2, and 3. Group B samples were tested for the presence of influenza B virus, human coronavirus OC43/HKU1, rhinovirus (HRV A/B), RSV B/A, and FLU A.

All PCR products were analyzed in 2% agarose gels by electrophoresis, stained with ethidium bromide and visualized on a UV light box. PCR products were compared with markers A and B bands in the gel to determine the size of the specimens tested, and to decide whether a swab was infected with a respiratory virus.

#### Viral culture and further typing

Sections of throat swab specimens were randomly selected and periodically shipped in an icebox to the Beijing Center for Disease Control and Prevention (CDC) for virus isolation and confirmation of pH1N1. Further isolation and sub-typing of FLU-A was performed in 190 samples. Virus isolation was processed using MDCK cell lines, and positive results were followed by the haemagglutination inhibition test using reference antisera (as recommended by the WHO manual: http://www.who.int/csr/resources/publications/influenza/whocdscsrncs20025rev.pdf). Once a negative or decreased reaction to the reference antisera occurred with the isolated virus, RT-PCR and sequencing was used to clarify the infection.

### Statistical Analysis

Data was entered into a database and analyzed using SPSS software version 16.0 (SPSS, Chicago, IL, USA). Continuously distributed variables were compared between FLU-A-positive ILI-cases and FLU-A-negative ILI-cases using the Student's t-test (t) if data was normally distributed and the Mann-Whitney U test if non-normally distributed. Categorical data or proportions were compared using Pearson's χ^2^ or Chi-square test. In order to evaluate the association between each variable and the FLU-A-positive cases at a univariated level, continuously distributed variables were re-categorised into binary variables, and univariate analysis using χ^2^ allowed calculation of odds ratios with 95% confidence intervals. P values<0.05 were considered statistically significant.

## Results

### Clinical features of the ILI case- patients

The clinical characteristics are summarized in Table 1. Of 279 ambulatory adult patients who met the ILI-case definition criteria, the median age was 31 years [Interquartile range (IQR) 26–44], with a range of 16 to 86 years, and 51.3% were males. A temperature reading >39°C was documented in 84(30.2%) of the 279 patients. Approximately 60% of patients reported cough, 62.8% pharyngalgia, 62.8% headache, 65.5% myalgia, and 2.7% diarrhea. Patients sought medical attention for the acute respiratory infection within a median of 1 day [IQR 1–2] after onset of symptoms. The median white cell count was 8.3×10^9^/l [IQR (6.2–11.2)×10^9^/l], and the mean percentage of neutrophils was 77.6% [95%CI (76.3,79.0)%].

### Clinical characteristics of ILI patients who tested positive for influenza A virus

Among the 279 patients tested by RT-PCR, 69 (24.7%) had influenza A virus and 13 (4.7%) had other respiratory viruses detected. Complete information concerning clinical presentation and routine examination was available for 190 patients (68.1% of the total), 61 of the FLU-A-positive cases and 129 of the FLU-A-negative cases. Data on the number of samples, clinical and univariate characteristics, P value and odds ratio (OR) (as well as the 95% confidence intervals (CIs)) in FLU-A-positive and in FLU-A-negative cases can be seen in Table 2. In comparison to FLU-A-negative ILI-case patients, cough was more likely to be reported in patients whose RT-PCR tested positive for influenza A virus (Table 2). Patients infected with FLU-A experienced more cough than patients infected with other viruses or those viruses free (χ2, p<0.001). Other clinical characteristics (such as sore throat, headache) appeared to be more frequent in those infected with the FLU-A virus than those non-infected, but the difference was not statistically significant. FLU-A-infected patients presented with a mean temperature of 38.6°C [95% CI (38.4, 38.7)] which on average, commenced 1.3 days before admission [IQR 1–2]. Approximately 30% of FLU-A-infected patients reported hyperpyrexia (temperature >39°C). The median age of patients who tested positive for influenza A was 32 years [IQR 27–47], and 44.3% were male. There was no significant difference in male and female rates. There was no difference in the neutrophil percentage between those whose RT-PCR tested positive for influenza A and those who tested negative. The clinical and univariate characteristics of influenza-A virus ILI-case patients are showed in Table 2.

**Table 2:** Characteristics of FLU-A and non-FLU-A ILI-patients in PKU People' Hospital betweenJune 2010 and May 2011.

Variables	FLU-A-positive ILI-case	FLU-A-negative ILI-case	OR	95%CI	p Value
	(n = 61)	(n = 129)			
Age(years)	32(27–47)	29(26–40)			0.087
Male sex	27(44.3%)	71(55.0%)	1.542	0.835 to 2.845	0.165
Temperature(°C)	38.6[38.4,38.7]	38.6[38.5,38.7]			0.433
Cough	48(78.7%)	67(51.9%)	3.417	1.691 to 6.904	0.000
Sore throat	41(67.2%)	78(60.5%)	1.340	0.706 to 2.543	0.369
Headache	41(67.2%)	76(58.9%)	1.430	0.754 to 2.710	0.272
Myalgia	38(62.3%)	84(65.1%)	0.885	0.471 to 1.665	0.705
Diarrhea	1(1.6%)	2(1.6%)	1.080	0.094 to 11.902	0.963
White cell count(×10^9^/l)*	8.0(6.4–9.9)(n = 56)	9.0(6.3–11.7)(n = 126)			0.061
Percentage of neutrophils*	77.8[75.4,80.1]	77.3[75.3,79.4]			0.782
Temperature(°C)>39°C	16(26.2%)	42(32.6%)	0.737	0.373 to 1.452	0.376
N%<70%*	8(14.3%)	26(20.6%)	1.560	0.658 to 3.701	0.310
Symptoms onset(days)**	1(1–2)(n = 57)	1(1–2)(n = 124)			0.190

*Total of 8(4.2%) participants missing information on routine examination results: 5 in the FLU-A-positive ILI-case group and 3 in the FLU-A-negative ILI-case group.

**Nine patients were missing perceived in the FLU-A-negative ILI-case group.

For RT-PCR-positive patients, we undertook telephone follow-up to collect further data (Table 1). In 20 FLU-A-positive cases, 4 (20%) received antiviral treatment within three days of the onset of symptoms. A further 21.2% (11 of 52) of these RT-PCR-positive patients had been vaccinated. A further 11.3% (8 of 71) reported chronic comorbid conditions (diabetes mellitus in four and hypertension in four). The median duration of fever was four days [IQR 3–5]. The median time for disappearance or abated of systemic symptoms in the presence of influenza virus infection, or other viral infections was 4 days [IQR 3–7] and 3 days [IQR 2–6] respectively.

### Temporal distribution of viral etiology

**Figure 1:**
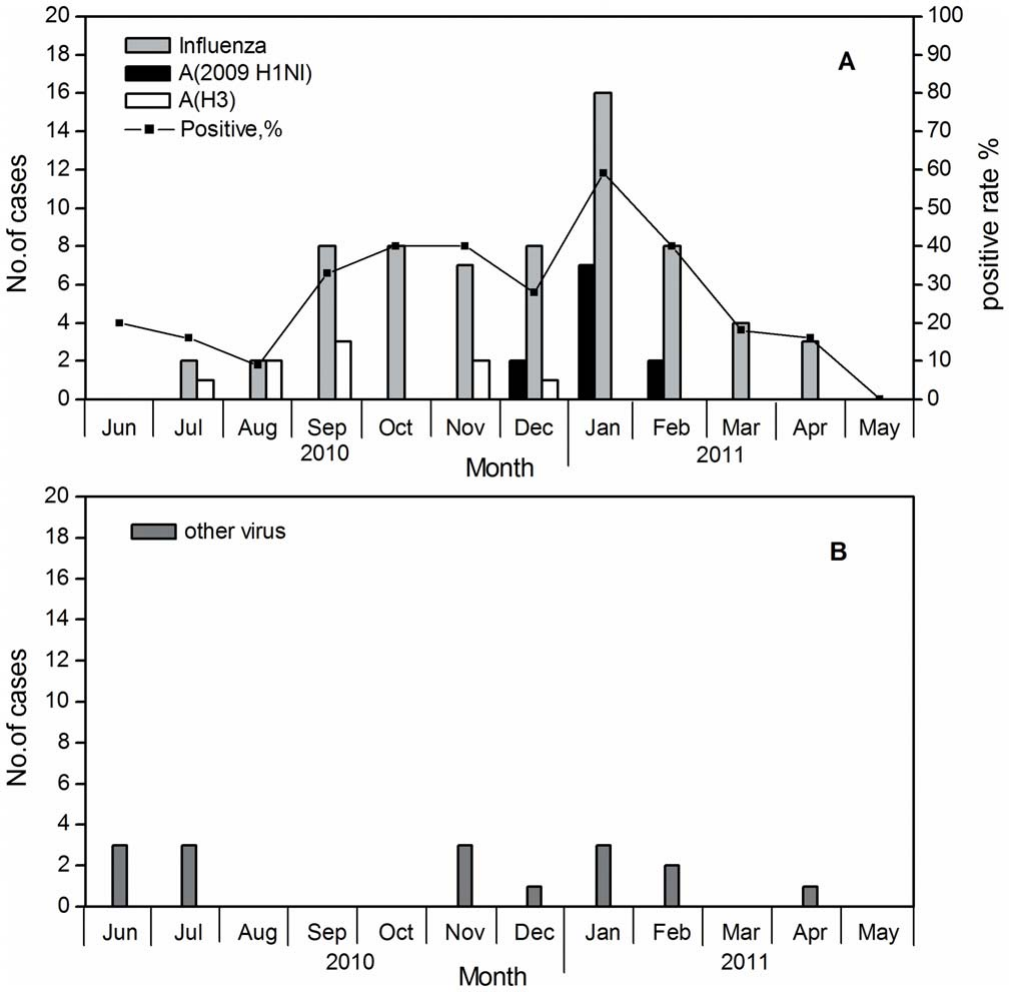
Monthly distribution of test-positive virus in the PKU People' hospital from June 2010 to May 2011. Figure 1A shows the number of respiratory specimens testing positive for influenza, by influenza type and positive rates of respiratory virus. Panel B shows the smaller number of other viruses. The rate of positive cases of the virus gradually increased from August to October, slowly decreased in November, and then increased again in January 2011. The peak of the positive rate occurred in January 2011 and influenza was the predominant virus between August 2010 and March 2011. Particularly in August, September and October 2010, and March 2011, all infections were caused by FLU only( Figure 1B). The number of positive cases of influenza virus increased rapidly in September and peaked in January 2011, following which it then decreased to normal levels in March 2011. Influenza A(H3) was the predominant viral etiological factor and was only observed from July to December 2010, and again, in January 2011, Influenza A(H3) were replaced by 2009 H1N1. 2009 H1N1 peaked in January 2011. Most influenza viruses were in fact influenza A, with influenza B virus only observed in April 2011. In June, multiple infections predominated. Each identified ILI was caused by a single virus except in June, July and November 2010 and January and February 2011: two FLU-A in August, eight FLU-A in September and in October each, eight FLU-A and one HRCV229E/NL63 in December, four FLU-A in March 2011, one FLU-A, two FLU-B and one ADV in April 2011, No virus was detected in May 2011. On the other hand, two of the three identified ILI samples in June were mixed infections, HRSV and HRV. Other double infections included: One HRCV/FLU-A in July, one FLU-A/HRV in November, two HRSV-A and FLU-A and one HRSV-A and HRV and FLU-A in January 2011, one HRSV-A and HRV and FLU-A in February 2011.

Among the 279 swabs collected, 82(29.4%) samples tested positive for at least one respiratory virus by multiplex RT-PCT. The temporal distribution of viral agents from confirmed cases tested by RT-PCR is shown in Figure 1. Influenza viruses (FLU) were the predominant viral etiology comprising 80.5% (23.7% of the total specimens) of the confirmed cases between June 2010 and May 2011 in Beijing. Almost all of these FLU-positive specimens were FLU-A cases, which accounted for 97.0%. Conversely, two influenza B virus-positive samples were identified among the samples. All other viruses were present at a frequency less than 3%: HRV and ADV in three cases individual (1.1% of 279); HRCV in two cases (0.7%); and HRSV in one case (0.4%). HMPV, FLU-C and HPIV were not detected, even thought the primers were designed to detect these strains. In addition, seven samples (2.5% of the total number of specimens) tested positive for more than one virus or were co-infections: HRSV-A and HRV in two cases; OC43 and FLU-A in one case; HRV and FLU-A in one case; HRSV-A and FLU-A in one case; and a combination of HRSV-A, HRV and FLU-A in two cases. The identity of each virus in these co-infections was confirmed by amplification with primers specific for each virus. Four of the eighteen patients aged 60 years or greater were positive for influenza A virus. For all detected H1N1-positive samples, no viral co-infections were observed.

Among the 279 swabs, 190 (68.1% of 279) samples were subtyped for 2009 influenza A (pH1N1) by the Beijing CDC. The number of respiratory specimens testing positive for influenza by influenza typing is shown in Figure 1. Of the 190 ILI case-patients tested, the positive rates for 2009 influenza A and influenza A(H3) were 5.8% (11/190) and 4.7% (9/190), respectively. Influenza A(H3) was only observed between July and December 2010, and was then replaced by pH1N1. Pandemic influenza A (H1N1) only tested positive in December 2010, and January and February 2011. According to the Chinese National Influenza Center(CNIC), influenza A (H3N2) viruses were the predominant viral etiological factors from June to early January in North China, and from late January, the 2009 influenza A(pH1N1) was the most prevalent, followed by H3N2 [4]. Few influenza B viruses (1.7%) were observed during the same period in North China [4].

### A reduction in the incidence of ILI

**Figure 2:**
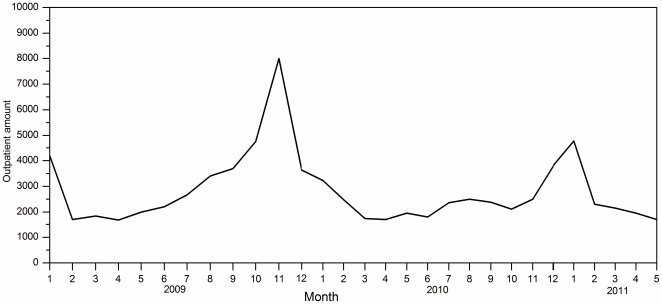
The adult outpatient service capacity of the Infectious Diseases Department of PKUPH, 2009–2011. In 2010, the overall number of outpatients was less than that of 2009, particularly between May and December. From May 2009, when pH1N1 emerged in Beijing, the capacity of the outpatient service of the Infectious Diseases Department of PKU People's Hospital increased rapidly. The number of outpatients peaked in November 2009, following which. The volume declined to reach normal levels in the third month of 2010. The numbers of outpatients was relatively stable in 2010, with an increase during November and December and a further peak in January 2011.

A decrease in the number of patients seeking medical attention was observed in 2010 (Figure 2). In 2010, the overall level of outpatient consultations was less than that of 2009, particularly between May and December. From May 2009, when pH1N1 emerged in Beijing [11], the capacity of the outpatient service of the Infectious Diseases Department of PKUPH increased rapidly. The peak in numbers attending the outpatient department occurred in November 2009. From then, the outpatient volume declined, reaching normal levels in the third month of 2010. The numbers of outpatients remained relatively stable in 2010. The volume again increased during November and December 2010, and peaked in January 2011.

**Figure 3:**
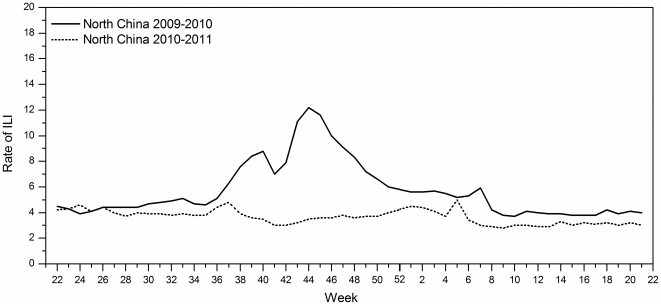
ILI rates in North China from June 2009 to May 2010 and June 2010 to May 2011. The weekly ILI rate increased rapidly in the 35^th^ week of 2009 in North China, with the highest weekly ILI rate of 12.1 cases per 100 consultations occurring in the 44^th^ week. However, the rate was relatively stable on the basis of the weekly ILI surveillance system of North China in 2010. The incidence rose slightly in January 2011 and the usual influenza seasonal peak appeared in the 5^th^ week of 2011. The percentage of patient visits for ILI peaked at 5.0% in the influenza 2010–2011 season. ^▴^The ILI rate of North China derived from data obtained from the Chinese National Influenza Center (CNIC).

In addition, a significant reduction in the percentage of outpatient consultations for ILI was detected during 2010–2011 in North China (Figure 3). During the 2009 pandemic, the highest weekly ILI rate was 12.1 cases per 100 consultations in week 44 of 2009 (Figure 3). The ILI rate was relatively stable, based on data available from the weekly ILI surveillance system of North China in 2010. The percentage of patient visits for ILI peaked at 5.0% in late January 2011.

## Discussion

RT-PCR is considered to be one of the most sensitive and specific tests for the diagnosis of influenza [12]. In addition, multiplex PCR methods can detect a panel of respiratory viruses or co-infections simultaneously [13]. In this study, pharyngeal swab specimens were collected from ILI case-patients, who strictly complied with Chinese CDC standards [3].

The general population in Beijing, China has been exposed to the novel pandemic H1N1 influenza virus since mid May 2009 [11]. According to the Chinese CDC, as of May 2011, 136869 confirmed cases and 875 deaths from pandemic influenza H1N1 2009, have been reported nationwide in mainland China [4], [14].

In this study, approximately 29.4% of the samples were positive for at least one virus, which is consistent with the results of other studies, in which between 0.9–27% [4], [15] and 44% [16] of reported samples were positive. Our research suggests that FLU-A was the predominant viral pathogen among ILI patients in Beijing from August 2010 to May 2011, which was similar to that of 2009, 2008 and 2006, but different to 2007 [3], [4], [17], [18]. Almost all positive samples demonstrated FLU-A strains, while few FLU-B strains were detected. This result differs from that reported from the United States, which demonstrated that 26% of the positive specimens were influenza B viruses [19]. This discrepancy may in part reflect the epidemic of influenza A virus in North China in 2010–2011. It is possible that FLU-B may not have caused ILI symptoms severe enough for the sufferer to seek medical attention. It is also possible that FLU-B was not circulating in this geographical area during this time. According to the CNIC, Influenza B virus-positive rate was about 1.6% in North China from June 2010 to May 2011 [4]. The percentage of tests that were positive for influenza which included FLU-A and FLU-B was 23.7% which was lower than the same period in 2009 [4], [17].

FLU strains, based on our data, accounted for approximately 80% of the RT-PCR positive cases, and in August, September and October 2010, and Mar 2011, all infections were caused by FLU-A alone. The incidence of FLU-positive specimens was high. This may reflect the possibility that our sample collection was biased towards patients exhibiting ILI, which is a clinical or symptomatic definition of influenza to identify potential influenza cases, in other word, influenza-like illness case-definition make influenza viruses as the virus most commonly detected [5], [18], [20], [21]. A study of human-to-swine transmission of pandemic influenza A virus concluded that the human ILI case definition has a high specificity and a low sensitivity for FLU-A [22]. Influenza viruses usually account for a much greater proportion of positive specimens of influenza-like illness in adults than other respiratory viruses during the peak seasons [23].

A total of 7 samples (8.5% of the total number of RT-PCR positive cases) revealed the presence of co-infections. In five FLU-A-positive samples, viral co-infections were observed, including one co-infection with HRCV in July, one with HRV in November, one with HRSV-A, and one with HRSV-A and HRV in January, and one with HRSV-A and HRV in February. A population challenged by multiple infectious agents may result in an epidemic and restructure of various viruses [24].

Rates of ILI are an indicator of trends for influenza pandemics [25]. Beijing is located in the temperate zone of the Northern Hemisphere, where influenza typically peaks seasonally once a year, and Beijing experience one peak of influenza activity and the peak occurred during December–January next year before 2009 [3], [26], [27], but ahead to November in 2009 [17], [26]. That the peaks in ILI and the increase in acute respiratory infections (ARI) are due to influenza is supported by the seasonal pattern of high-probability ILI, the low level of respiratory syncytial virus infections, and laboratory results in the influenza season [23], [28], [29]. During the 2010–11 influenza season, a seasonal pattern in ILI activity was observed and influenza activity peaked in late January 2011 in Beijing. Compared with the previous pandemic year (2009), lower outpatient numbers were observed during 2010–11(Figure 2). Overall, the rates of influenza-like illnesses in outpatients were lower during the 2010–2011 season, than during the 2009–10 pandemic influenza season (Figure 3).

As a result of the requirement for fever in our definition of ILI, our calculated incidence may underestimate the true incidence of ILI in the cohort. Our study shows that the positive rate of influenza virus was consistent with changes in the ILI rate during the same period.

More than 70% of ILI-case patients were infected with p-H1N1 between June 2009 and January 2010 [4]. Compared to the previous year, the age of FLU-A patients ranged from 18 to 61 years with a mean age of 36.7 years, which is older than the age of 2009 ILI-case patients (mean age was 23.4 years) [11].

In 197 (70.6%) specimens, no viral etiology was identified or the virus was not detected. This may reflect the fact that only viruses known to cause respiratory symptoms were tested, and therefore the remainder may have been caused by other respiratory viruses or by other micro-organisms, which could also have been additional pathogens in the positive specimens [5]. It is also possible that some viruses could not be detected due to low levels of shedding.

In this study, we attempted to identify symptoms associated with influenza A infections. However, it is widely known that a clinical diagnosis of influenza is not straightforward, and it is difficult to find symptoms or combination of symptoms specific for influenza [20], [30]. We found that cough was significantly associated with influenza A (χ2, p<0.001). This is consistent with the results of Boivin, of Ohmit and of Monto, who reported that cough and a fever>38°C were associated with a positive PCR test in the influenza population, when influenza was prevalent within the community [7], [31], [32]. Our specimens were collected during the influenza season in Beijing and the cough appears to be specific for influenza in this cohort. This is also consistent with the view of Call et al, who believe that both fever and cough are more specific for influenza among elderly individuals when influenza virus is circulating in an area [30]. We did not find any statistically significant difference in the occurrence of fever (temperature >39°C) between the FLU-A-positive group and the FLU-A-negative group (χ^2^, p>0.05). A study that involved all age groups demonstrated that muscle and joint pain and headache were associated with influenza [20]. However, we did not find any statistically significant differences in the occurrence of muscle and joint pain, and headache between the FLU-A-positive group and the FLU-A-negative group (χ^2^, p>0.05).

This study has limitations. No testing for other etiologies of acute respiratory illness was performed. As is generally known, respiratory viruses, bacteria and other micro-organisms can cause respiratory illness with influenza-like symptoms [5]. Without doubt, other micro-organisms could have been additional pathogens in the positive specimens.

In conclusion, pH1N1 did not affect typical influenza seasonal peaks, although FLU-A remained the predominant virus in Beijing in 2010. Symptomatically, cough was associated with FLU-A infection. The positive rate of influenza virus was consistent with changes in the ILI rate during the same period and there was a significant reduction in the incidence of ILI in 2010 compared to 2009. The findings of this study may facilitate the clinical discrimination of influenza A virus infection, as well as providing data and distribution information for virologic surveillance of influenza.
